# Medical News and Miscellany

**Published:** 1890-12

**Authors:** 


### Medical News and Miscellany.

Dr. T .J. Everett has removed from South Bosque to Lorena.

Dr. T. P. Davis, of Canton, Texas, is taking a special course at Atlanta, Ga., Medical College.

Dr. R. P. Talley, of Belton, will shortly remove to Temple—the great centre of railroad crossings.

Christmas is here again. Now is the time to remember the poor—editor. Send in your two dollars—Christmas gifts—for the JOURNAL.

Dr. A. V. Doak, of Taylor, has made a fortune in practice and real estate and has given notice that after 15th instant he will retire frm practice.

The Journal has received Blackiston's Physicians' Visiting List for 1891. It is the standard, and has stood the test of thirty-eight years trial by the profession, and still holds the lead.

**Dr. L. W. Cock** has left Laredo—he says he and the boom left together—and is temporarily at his old home in San Marcos—catching bass—and looking around for a new location to practice,

---

**The Business Men** of Galveston have memorized Governor-elect Hogg, asking that Dr. R. M. Swearingen be appointed State Health Officer. This is a supplement to the physicians' memorial already mentioned.

---

**The Journal** has recently been favored with calls from a number of prominent physicians—amongst them Dr: W. W. Reeves, of Wills Point; Dr. T. A. Pope, of Cameron; Dr. R. P. Talley, of Belton; Dr. F. S. White, and others.

---

**In connection** with the appointment of State Health Officer we omitted to mention in the list of aspirants the names of Dr. C. T. Simpson, of Temple, First Vice-President T. S. M. A.; Dr. W. W. Reeves, of Wills Point, and Dr. W. L. Barker, of Waco.

---

**Dr. James Kennedy**, of San Antonio,—the newly elected Secretary of the West Texas District Medical Society,—is one of the best practical chemists in the State, and should Texas have a State Board of Health, no better chemist could be found to serve on it.

---

**Food for Infants.**—Dr. Louis Starr recommends the following as the best substitute for the mother's milk in *gradual* weaning of a child, say at ten months; it may also be employed to support the breast when the mother's milk is insufficient:

Cream ℥ss.

Milk ℥ii.

Sugar of milk ʒss.

Water ℥i.

Should the quantity fail to satisfy the child, all the ingredients except the cream may be increased until the mixture measures six, eight or twelve ounces.—*Journal of the American Medical Association.*

Married—in Austin, Texas, Dec. 15, 1890, Dr. C. T. Young, of Waco, to Miss Jennie Duffield, of Austin.

Married—in Austin, Dec. 1, 1890, Mr. R. G. Johnson, of Fort Worth, to Miss Maud, daughter of Dr. T. D. Wooten, of Austin.

You can get the New Orleans Medical and Surgical Journal ($3) for $2 by taking it in connection with this JOURNAL—both for $4.

The Texas Wholesale Drug Social Society is the name of an organization effected at Fort Worth, Dec. 14, by the wholesale drug men of Texas. B. F. George, of Houston, was elected President, and W. N. McKnight, of Fort Worth, Secretary.

Married.—On Wednesday evening, at 7:30 o'clock, at the residence of the bride's father, Mr. N. B. Steddum, in Mt. Calm, Dr. J. B. Creswell and Miss Minnie Steddum were united in the holy bonds of matrimony. Rev. Mr. Lenz officiating.—*Hubbard City News, Dec. 4.*

Removals.—Dr. St. Cloud Cooper has removed from Jefferson to San Antonio. Dr. W. H. Wilson has removed from Weldon to Trinity. Dr. F. M. Leonard has removed from Brookston to Harold, Wilbarger county. Dr. A. N. Carr has removed from Ragsdale, Fannin county, to Greenville, Hunt county.

Dr. F. S. White, the able First Assistant Physician of the State Lunatic Asylum at Terrell, was in Austin lately. The Doctor has had a fine experience in asylum administrations and practice, having filled his present position about nine years. He is prominently spoken of as the coming superintendent for the new asylum at San Antonio, now in process of construction,—a position for which his training at Terrell, his special studies, and his natural and acquired abilities admirably fit him.

Trades and Occupations.—*The Youth's Companion* for 1891 will give an instructive and helpful Series of Papers, each o which describes the character of some leading Trade for Boys or Occupations for Girls. They give informatiou as to the Apprenticeship required to learn each, the wages to be expected, the qualities needed in order to enter, and the prospects of success. To New Subscribers who send $1.75 at once the paper will be sent *free to Jan. 1, 1891, and for a full year from that date.*

Address, THE YOUTH'S COMPANION, Boston, Mass.

---

**News from Berlin—Koch's Remedy.**—Through the kindness of a gentleman in Austin who has been in correspondence with Dr. Koch the JOURNAL is furnished with the following extract from a recent letter from the great bacteriologist. Dr. Koch says: "If the lymph be given to every applicant pretending to be a doctor, or the preparation of same, it would perhaps cause more deaths than now this dread disease, consumption; and we can only give it to institutions of known repute where they will earnestly experiment, or to recognized regular physicians. We have had no applications from Texas as yet, and could not fill them at present if we had, but will be able to do so in a short time, to those who may be desirous to properly help us in our experiments. We have received ample means from the government to produce the lymph, and it can be given to applicants at 25 mark for 5 grammes. One vial contains 5000 injections."

---

**We are requested** by Mr. Jos. A. Hincks, Secretary of the City Eye, Ear, Nose and Throat Free Hospital, of New Orleans, La., to make the following announcement to the medical profession:

On the first Monday in January, 1891, the Executive Committee of said Institution will proceed to elect, upon the recommendation of the medical staff, two resident surgeons, upon the following conditions:

1. They must be graduates of a reputable medical college.
2. They must be under thirty years of age.
3. They must sign an agreement to serve for one year.

The hospital will furnish only lodging and its unsurpassed facilities for the study of the eye, ear, nose, and throat.

For the information of those interested, we will state that during a period of eleven months 32,712 consultations were given to 3206 patients, and over 500 operations of all kinds performed.

Applicants should state what other degree they possess besides their medical degree. References should also be given.

Appllcations should be in the hands of the Secretary by Dec. 25, 1890.

---

**Extracts from recent German Publications.**—The following excerpts in regard to Dr. Koch and his lymph are taken from recent German publications:

"The Emperor of Germany has presented Dr. Koch with the Cross of the Red Eagle, and it is the first time this honor has been conferred upon one who is not in possession of the minor honors."

"Dr. Stellwag, of the Vienna University, warns his scholars 'not to believe what they hear of Dr. Koch's remedy, but only believe what he himself positively asserts. As yet nothing is definite but that it will cure lupus. It is not positively ascertained whether lupus is originated by the bacillus which causes lung tuberculosis.' "

"Dr. Kraus, of Vienna, who attended experiments of Koch's injections at Berlin, says that he believes in its efficacy in muscle and skin tuberculosis but has doubts about it regarding the cure of advanced lung tuberculosis."

"Dr. Ullmann, a renowned authority in bacteria, says it will take a full year of experiments and treatment in proper institutions to ascertain the fact whether in the incipient or advanced stage of lung tuberculosis the lymph will be a curative agent."

"Dr. Kowalski, chief of the Bacteriologic Institute of Vienna, defends Dr. Koch in his secrecy of the ingredients or make-up of the lymph, and says it is the strongest medicine now known in our materia medica and one that can not be too guardedly experimented with."

**Mosquera's Food Products—Beef Meal, Beef Cacao.**—Parke, Davis & Co., whose reputation for original work has long been established, announce that after thorough study of the various food products they can now supply preparations which will fulfill all the requirements for therapeutic and dietetic use.

Physicians, in their practice, very frequently meet with cases where nutrition is of more importance than medication; in fact, cases where nutrition is the only agent they can count upon. The question of replacing the waste of tissue, where normal nutrition is inefficient, by means of concentrated or predigested foods, is one that always presents many difficulties, there being very few preparations, if any, that meet all the requirements of the medical profession.

Heretofore medical practitioners have had at their disposal a great variety of preparations of meat. These are divisible into four great classes. We have, in the first place, the extractr of meat, prepared after the formula of Liebig; then the meat juices; next the ordinary powdered meats; and, finally, the meat peptones.

The ordinary process of preparing meat extracts involves a simple extraction of meat with either warm or cold water, and an evaporation of the resulting solution continued until reduced to a thick liquid or paste. This extract contains the inorganic soluble salts of the meat and some stimulating organic matter, but none of the nourishing, flesh-forming albuminous substance.

The meat juices are usually cold extractions of the meat, and such products contain some soluble albumen, which coagulates out upon boiling, and, naturally, cannot amount to much more than four or five per cent. The meat juices, therefore, possess but little nutritive value.*

Powdered meats, as heretofore known, are nothing more nor less than the residue left after extracting all the soluble constituents. Dujardin Beaumetz and several therapeutists, as a result of a careful line of experiments, concluded that this powder possessed a high nutritive value, and could be employed to advantage in the treatment of certain diseases (consumption and dyspepsia especially). That they are concentrated nutrients is a fact, for beef, in its natural condition, contains 75 per cent. of moisture, all of which is driven off in the preparation of the powder.

---

[Wyeth's Meat Juice is an exception and possessess real value.—ED.]

The fact, however, that these powders are liable to become ran cid, or else have been deprived of the inorganic salts, peculiar to meat in its natural state, which salts are quite essential in the digestive process, is an objection to the meat in this form. Moreover, powdered beef requires just as much effort on the part of the stomach to digest it, as does ordinary beef, and for this reason cannot be regarded as a proper food for patients suffering with derangement or weakness of the digestive organs.

Another group of meat preparations embraces the meat peptones.

Peptone is the ultimate product of digestion, and the form in which the albuminous or proteid matter is assimilated by the system. These peptones are invariably the product of the artificial digestion of meat by animal pepsin and hydrochloric acid, or, although to a smaller extent, by the digestive ferment of the carica papaya. These are the only preparations really valuable as nutrients. But the physician meets here with another difficulty, in many cases insurmountable; the taste of the peptones is, more or less, bitter and objectionable to the palate, so that patients either absolutely refuse to take them, or take them only with the greatest repugnance. Besides this, their price is comparatively so high that frequently the physician is obliged to abstain from prescribing them.

*All the difficulties heretofore encountered by the medical profession in the use of predigested foods, have been overcome by the new food products of the Mosquera-Julia Food Company.*

Mosquera's Beef Meal contains all the stimulating principles of the extracts of meat, and, in addition, the nutritive principles which the extracts lack; all the albumen of meat juices without their weakness; all the strength of powdered meats without their rancidity and insolubility; all the peptone of the peptonized meats without their bitterness.

The claims we make on behalf of Mosquera's Beef Meal, therefore, cannot be over-estimated; they are based on its analisis and properties, and may be condensed as follows:

Mosquera's Beef Meal is a perfectly pure predigested meat, containing all the nutritious constituents of good lean beef, half of which are in soluble form, ready for immediate assimilation, and the other half easily digestible by the gastric and pancreatic juices. Therefore the entire preparation, being practically dry, is composed of nutritive matter, containing about 40 per cent. of soluble peptone and albumose.

It represents, in actual nutritive value, at least six times its weight of good lean beef.

It is perfectly palatable, and will be tolerated with ease by the most delicate stomach.

It admits of being admininistered in a variety of forms, thus avoiding monotony in the food.

It is the most nutritious as well as the most economical concentrated food.

It must be understood that Mosquera's Beef Meal is not a ready prepared dish, but rather a raw product. It is nothing more than a concentrated beef, converted by artificial digestion into a form which renders it assimilable upon mere contact with the mucous membranes of alimentary canal. It, therefore, must be treated by the nurse or cook with the same regard to flavor and taste they would exhibit in the preparation of beef steak. Ordinary beef, if simply boiled in water, would neither yield a palatable buillon nor be eaten itself; salt or other condiments must be added to it. So also, in the use of this beef meal, ingenuity has necessarily to be exercised in its preparation. No matter how palatable or nutritious a food may be, unless presented in a variety of forms it will inevitably become monotonous and even repulsive, this being especially true with patients whose digestive organs are in a weak and debilitated condition. If, therefore, a patient is to take the beaf meal for a length of time, it must be administered in a variety of forms to insure the benefit of all its nutritious value.

It may be given in different soups, condimented to suit the taste of the patient, as also mixed with buscuit powder or oatmeal porridge and milk and sugar. Again, it may be mixed with chocolate, which makes a delicious beverage, or given in the form of a sandwich, and finally as a plain beef tea, simply dissolving it in hot water, adding salt.

Mosquera's Beef Cacao consists of equal parts of beef meal, sugar and a superior article of Dutch cacao. It does not require cooking, but may be mixed with warm milk exactly like ordinary chocolate, and so completely is the taste of beef disguised that it cannot be detected. Requiring therefore no previous preparation it is most conveniently administered.

To physicians interested a pamphlet fully descriptive of the special advantages, uses and methods of administration of these preparations will be mailed on request, and samples will be sent to physicians who desire to clinically test them in practice.—*Exchange.*

---

**Notes Upon Somnal, the New Hypnotic.**—The following notes upon Somnal, by Frank Woodbury, A. M., M. D., Fellow of the College of Physicians of Philadelphia; Hon. Professor of Clinical Medicine in the Medico-Chirurgical College, are taken from a reprint of *The Dietetic Gazette*, July number:

"Last fall Radlauer, of Berlin, brought to the notice of the medical profession a new compound to which he gave the name of Somnal, in acknowledgment of the remarkable hypnotic properties which it appeared to possess. It was formed by the union of chloral, alcohol, and urethane, according to the original notice, but is not a simple mixture of these bodies. It differs from chloral-urethane by the addition of $C_2H_4$, its formula being $C_7H_{12}Cl_3O_2N$. The method of manufacture is by direct combination of chloral alcoholate and urethane in a vacuum apparatus, according to its discoverer, who states that its composition might be graphically represented thus:

$$\begin{array}{l} \qquad\qquad\quad OC_2H_5 \\ CCl_3—C—H \\ \qquad\qquad\quad NHCOOC_2H_5. \end{array}$$

Specimens of this new hypnotic having, through the courtesy of Messrs. Eisner & Mendelson Co., been placed in my hands for examination and trial, I will here very briefly communicate some of the results thus far obtained, reserving my final judgment upon the drug until experience has been more extended.

*Physical Characters.*—Somnal is a colorless liquid, resembling chloroform in its appearance and behavior when added to cold water, in which it forms globules and refuses to mix or dissolve. When shaken with water, the mixture is milky but quickly separates. It is soluble in hot water and alcoholic solutions, and dissolves resinous substances and fats. The odor is faint, not very penetrating or disagreeable, and resembles that of the spirits of nitrous ether, or re-christallized chloral. The taste is very pungent ; and for administration it needs free dilution. It may be given with whiskey or solution of tincture of zingiber or syrup of licorice. Somnal is inflamable, burning with an alcoholic flame; it does not evaporate quickly, and leaves a greasy stain upon blotting paper. Specific gravity greater than water; reddens litmus paper slightly.

*Physiological Effects.*—In its action it resembles chloral in quickness of effect and naturalness of the sleep produced. No marked depressing influence was exerted upon the pulse or respiration rate, though it was noticed that the breathing became slower and the pulse slower and fuller as in natural repose. No disagreeable after-effects. The head was clear and the stomach was unaffected; the patients generally had an appetite for breakfast. No constipating effect. The kidneys acted rather more freely than usual. My colleague, Dr. Earnest Laplace, to whom I gave some of the drug for trial at the Philadelphia Hospital, writes as follows:

"I have given Somnal a fair trial upon six patients at the Philadelphia Hospital. In no case were the patients told what was given them, so outside of the bare possibility of the patients' falling asleep through natural causes, somnolence was brought on by the drug. It was administered in a solution of tinct. zingiberis in half-teaspoonful doses, and was found palatable.

"Administered at 4 p. m., at a moment when patients were not generally asleep, in four cases sleep came on within half an hour, which lasted from four to eight hours; the two other cases showed no effect from the drug. It is their habit to get at least gr. ¼ of morphine sulph. to put them to sleep every night, as they are sufferers from intractable malignant growth.

"In no case was there any noticeable after-effect.

"I have not formed any opinion upon the length of time that the drug could be used daily upon the same patient.

"To this I might add that no depression of the normal temperature was noticed in any case in my hands, and thus far I have not used it in pyrexia."

*Therapeutic Application.*—The effects of Somnal in producing natural sleep suggested its use in insomnia. The first case in which I used it was in a patient suffering with acute alcoholism, who had been under treatment for a fortnight in an institution where he had a free supply of liquor, and he came out rather worse than he went in. He was thirty-nine years of age, very tremulous, and could not sleep, or if he dozed off, would immediately wake up. I gave him, about 3 p. m., thirty minims of somnal (or rather a drachm of a mixture of equal parts of somnal and whiskey), well diluted, and went into an adjoining room to speak to an attendent. Upon my return I was surprised to find him fast asleep, although I had not been away from him more than fifteen minutes. He slept for four hours, and then was able to take something to eat. At ten o'clock he kad another dose and he slept until seven the next morning, having waked up once only during the night and insisted upon having another dose, and immediately after taking it he fell asleep again. The next night he was given a double dose at 10 p. m., and he slept all night without waking. No bad effects were observed. The somnal was given for four nights, when he was so nearly well that it was suspended, as he had had good natural sleep at night and seemed quite restored. Alcohol was positively prohibited, the only substitute allowed being Elixir of Coca and Camellia (P. D. & Co.) in tablespoolful doses; in which it is true there was a small amount of alcohol, which was quite infinitesimal when compared with what he had been using. Somnal, therefore, acts well as a hypnotic in acute alcoholism as a tranquilizer and hypnotic.

In a case of neuralgia of the bowels (visceral neurosis of Allbutt), where the patient had a sleepless night, a dose of twenty minims relieved nausea and pain, and the patient fell asleep.

In syphilitic headache and insomnia, somnal in moderate doses failed to produce sleep, which was afterwards secured by potassium bromide and iodide, and antipyrine.

In cases of insomnia, fretfulness, and restlessness in young children, somnal with mint water and syrup offers better results than opiates, and is much safer. The same remark probably applies to the use of somnal in acute pneumonia, but I have not been able to confirm this yet by actual trial.

Without further going into detail it may be stated in conclusion that somnal acts as a hypnotic, but instead of depressing the system as chloral does, it slightly stimulates the gastric mucous membrane, relieves nausea and pain, improves the appetite, increases secretion (probably), does not cause constipation. The circulation, respiration and temperature are not notably depressed after its administration. No disagreeable after-effects have been observed. As it is rapidly eliminated from the body it may be administered each night for a number of days without any obvious ill effects. It acts very much like chloral, but is more pleasant to take and not so depressing in its effects upon the nervous system and the circulation.

---

Now is the time to subscribe for the JOURNAL. Always bright and newsy, and only two dollars a year.

Perspiration of the Feet.—

℞. Glycerine,............1 oz.
Kennedy's Ext. Pinus
Can...............1 oz.
Aquæ..............2 oz.
Essence of Bergamot ..2 drachms.

Mix and apply twice each day. The results are surprisingly rapid and happy.

